# Impact of Concurrent Ischaemic Stroke on Unfavourable Outcomes in Men and Women with Dilated Cardiomyopathy

**DOI:** 10.31083/j.rcm2506215

**Published:** 2024-06-14

**Authors:** Zexin Fan, Chao Wu, Chaobin Wang, Chun Liu, Libo Fang, Lin Ma, Wenlong Zou, Boyi Yuan, Zeyu Ji, Bin Cai, Guangzhi Liu

**Affiliations:** ^1^Department of Neurology, The Second Hospital of Shanxi Medical University, 030001 Taiyuan, Shanxi, China; ^2^Department of Neurology, Beijing Anzhen Hospital, Capital Medical University, 100029 Beijing, China; ^3^Department of Neurology, Beijing Fangshan District Liangxiang Hospital, 102401 Beijing, China; ^4^Department of Neurology, Mechinka Hospital, Dnipro State Medical University, 49044 Dnipro, Ukraine; ^5^Department of Neurology, Beijing Fuxing Hospital, Capital Medical University, 100038 Beijing, China

**Keywords:** ischaemic stroke, dilated cardiomyopathy, sex, risk factor, neurocardiology

## Abstract

**Background:**

Growing evidence suggests that concurrent ischaemic stroke (IS) exacerbates the prognosis of patients with dilated cardiomyopathy (DCM) and that this effect may be further influenced by sex. However, the exact effect of sex remains unclear. This study aimed to explore the effects of the relevant risk factors on the prognosis of patients with DCM and concurrent IS. Considering the sex differences in DCM, this study further investigated the impact of concurrent IS on the prognosis of men and women with DCM.

**Methods:**

A total of 632 patients with DCM enrolled between 2016 and 2021 were included in this study. Clinical data were obtained from medical records, and all participants were followed up in the outpatient clinic or by telephone for at least 1 year. A Cox proportional hazards model and Kaplan–Meier curves were used to evaluate the effects of concurrent IS on the prognosis of patients with DCM.

**Results:**

Patients with DCM complicated with IS (DCM-IS) had significantly lower cumulative survival rates than patients with DCM without IS (non-IS) (74.6% vs. 84.2%, χ^2^ = 6.85, *p* = 0.009). Additionally, IS was associated with greater risks of death and heart transplantation (HTx) in men (75.8% vs. 85.1%, χ^2^ = 5.02, *p* = 0.025), but not in women (71.0% vs. 81.5%, χ^2^ = 1.91, *p* = 0.167).

**Conclusions:**

This large-scale multicentre prospective cohort study demonstrated a poorer prognosis in patients with concurrent DCM and IS, particularly among men. Patients with DCM should not be overlooked in IS screening, emphasis should be placed on the occurrence of IS in patients with DCM. Early and proactive secondary prevention of cerebrovascular diseases might improve the prognosis of DCM patients. More intervention studies focusing on men with DCM complicated with IS should be prioritised.

## 1. Introduction

Dilated cardiomyopathy (DCM) is a spectrum of heterogeneous myocardial disorders characterised by ventricular dilation and depressed myocardial performance that is not explained by hypertension or valvular, congenital, or coronary artery disease [1]. Genetic mutations, infections (viruses, bacteria, protozoa, helminths, and fungi), autoimmunity, toxin exposure, metabolic or endocrine dysfunction, and neuromuscular diseases have been gradually recognised as diverse aetiologies of DCM after decades of research [2]. DCM typically occurs in the 3rd or 4th decade of life; its main clinical manifestations include progressive cardiac enlargement, reduced ventricular systolic function, heart failure, ventricular and supraventricular arrhythmias, conduction system abnormalities, thromboembolism, and sudden cardiac death [3, 4]. Growing evidence suggests that sex differences may exert a crucial influence on cardiovascular disorders, including DCM. A population-based study, despite its limited data owing to a small sample size, revealed higher expression of apoptosis-associated proteins in men with acute DCM, significantly correlating with a lower left ventricular ejection fraction (LVEF) [5]. Moreover, several animal studies have reported a heightened susceptibility to cardiac remodelling and DCM in male animals [6, 7, 8, 9], further underscoring the impact of sex differences on the pathophysiology of DCM.

Thromboembolism represents a severe and potentially catastrophic complication of DCM, with the incidence rate of embolic events ranging from 1.5 to 3.5 per 100 patient-years in patients with DCM [10], including ischaemic stroke (IS). Young adult patients presenting with concurrent IS (i.e., individuals with a history of IS before enrollment) and DCM face a bleak prognosis, despite their initial IS severity being relatively mild at admission [11]. Conversely, a retrospective study of 20 patients with cardioembolic stroke experiencing either hypertrophic cardiomyopathy or DCM revealed that 40% of patients with cardiomyopathy developed stroke, with 38% succumbing within 5 years, even though they were all aged <60 years [12]. Additionally, DCM itself carries an unfavourable prognosis for patients with LVEF <35%, right ventricular involvement, and New York Heart Association (NYHA) functional class III or IV [13]. However, the role of sex remains enigmatic because of the paucity of cohort studies involving patients with DCM and concurrent IS. Consequently, this study aimed to investigate the impact of concurrent IS on the prognosis of DCM patients of different sexes.

## 2. Materials and Methods

This was a population-based multicentre prospective cohort study. The included participants were inpatients at three tertiary medical centres between January 2016 and December 2021. All patients were enrolled at the time of first hospitalization and underwent follow-up either in the outpatient clinic or via telephone for at least 1 year. This study was conducted in accordance with the principles of the Declaration of Helsinki [14, 15]. This protocol received approval from the Ethics Committee of the Beijing Anzhen Hospital of Capital Medical University (2023176X). Written informed consent was obtained from all participants.

The inclusion criteria were (1) age ≥18 years; (2) diagnosis of DCM according to the European Society of Cardiology proposal based on systolic dysfunction and left ventricular dilatation determined by echocardiography or cardiac magnetic resonance imaging (MRI), after excluding abnormal loading conditions or coronary artery disease: LVEF ≤45% or left ventricular fractional shortening (LVFS) <25%, left ventricular end-diastolic dimension (LVEDD) >5.0 cm (female) and >5.5 cm (male) [16]; and (3) NYHA functional class ≥Ⅱ. The exclusion criteria were as follows: (1) ischaemic heart disease, rheumatic heart disease, arrhythmogenic cardiomyopathy, congenital heart disease, pulmonary heart disease, drug-induced cardiomyopathy, hypertensive heart disease, perinatal cardiomyopathy, valvular heart disease, or alcoholic cardiomyopathy; and (2) missing clinical data.

All clinical data were obtained from electronic medical records. The diagnosis of IS relied on medical histories, clinical examinations, brain MRI scans, and magnetic resonance angiography results, as confirmed by two attending neurologists [17]. Data from the first admission were collected to analyse individuals with multiple hospital admissions. The DCM patients were categorized into two groups based on whether they had IS before enrollment: the DCM with IS group (DCM-IS) and the DCM without IS group (non-IS). In patients with both DCM and IS, only data from the onset of the first IS were used in this study. The primary outcome was a composite of heart transplantation (HTx) and all-cause death, including death caused by cardiovascular events, IS complications, and other causes during follow-up.

Statistical analyses were conducted using SPSS version 25 (IBM Corp., Armonk, NY, USA). In detail, clinical data included personal history, past medical history, clinical laboratory test, echocardiography reports, drug use of angiotensin-converting enzyme inhibitor (ACEI) or angiotensin receptor blocker (ARB) or angiotensin receptor-neprilysin inhibitor (ARNI) or β-Blockers or anticoagulation, and history of implantable cardioverter-defibrillators (ICD) or cardiac resynchronization therapy (CRT). Data (including systolic blood pressure and leukocyte), which followed a normal distribution, were presented as the mean ± standard deviation (SD) and were analyzed using Student’s* t*-tests. Data (including age, haematocrit, platelets, haemoglobin, estimated glomerular filtration rate (eGFR), serum Na+, hypersensitive C-reactive protein (Hs-CRP), D-dimer, LVEDD, LVEF, pulmonary artery pressure, and left atrium diameter), which followed a non-normal distribution, were presented as the median (interquartile range) and were analyzed using Mann–Whitney *U* tests. Categorical variables were presented as frequencies (percentages, %) and were analyzed using the chi-squared test.

Furthermore, this study divided the DCM patients into two groups (yes vs. no) based on whether patients with DCM experienced all-cause deaths or underwent HTx within 1 year. Univariate Cox analysis was applied to identify the potential factors, including personal history, past medical history, clinical laboratory test, echocardiography reports, drug use of ACEI/ARB/ARNI/β-Blockers/anticoagulation, and ICD/CRT, in the associations with the DCM’s prognosis. Multiplicative interaction terms (IS*sex) were used in multivariable-adjusted Cox models to explore the moderating effects of critical confounders. Subgroup analyses were conducted if there existed possible strata effects (*p* for interaction <0.1). Variables with statistical significance in univariate Cox analysis were further included in the multivariate Cox proportional hazard model to assess the robustness of results. Finally, survival curves were plotted using the Kaplan–Meier method, and differences were compared using the log-rank test. *p*-values < 0.05 were considered statistically significant.

## 3. Results

**Fig. 1.**
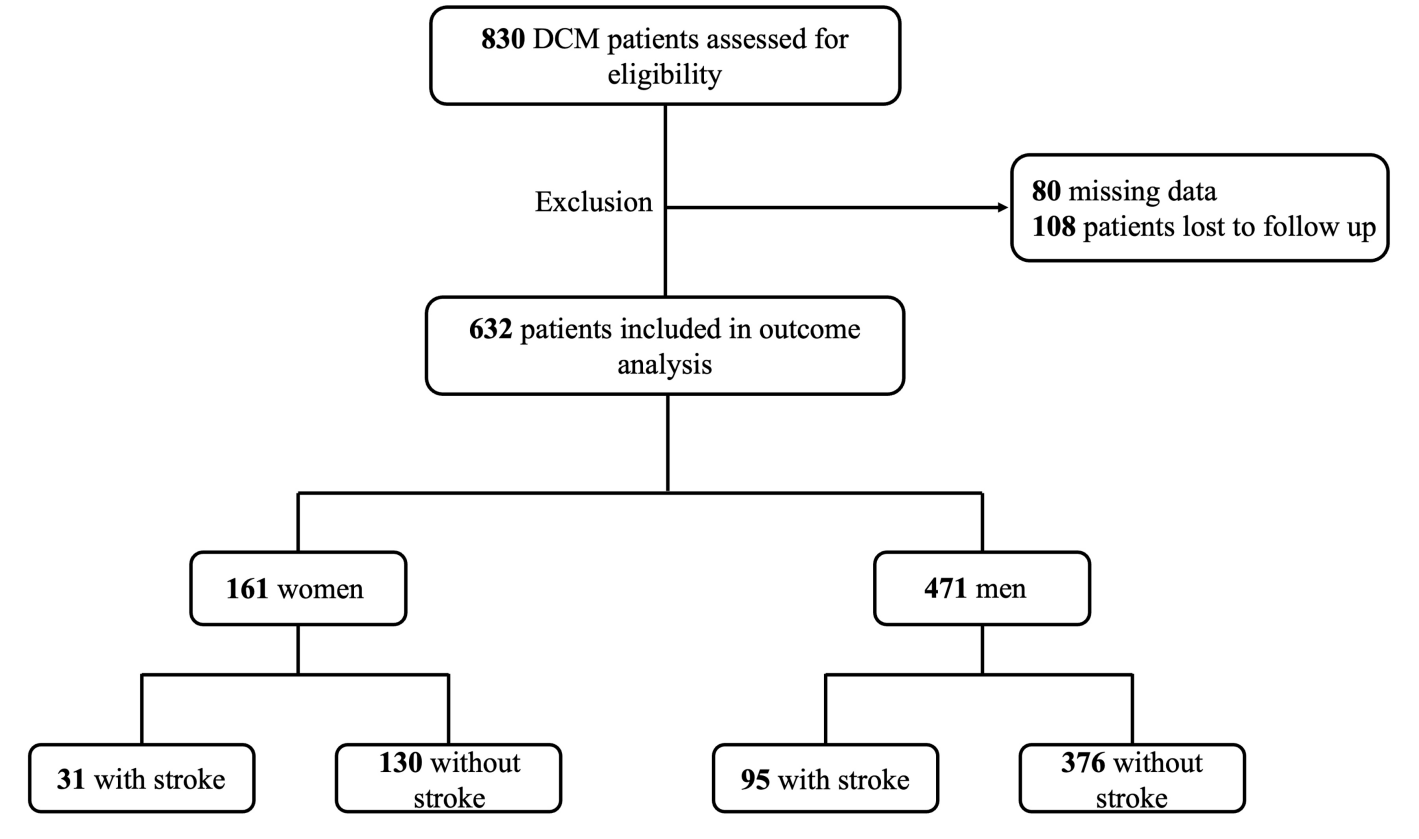
**Flowchart describing the enrolment of patients with DCM complicated with IS. **DCM, dilated cardiomyopathy; IS, ischaemic stroke.

Among the 830 patients with DCM, 632 were ultimately enrolled in this study, comprising 126 patients with IS and 506 patients without IS (Fig. 1). The median time between IS and the start of the study was 6 (interquartile range [IQR]: 1–12) months. The baseline characteristics of these patients, as stratified by sex, are summarised in Table 1. Female patients with DCM-IS exhibited less severe heart failure, higher Hs-CRP levels, and higher incidence of intracardiac thrombosis compared to their counterparts without IS. Moreover, significant differences in hypertension, hyperlipidaemia, atrial fibrillation (AF) or atrial flutter, eGFR, serum Na+, D-dimer, and Hs-CRP levels, as well as intracardiac thrombosis, were observed between male patients with and without DCM-IS.

**Table 1.** **Comparison of baseline data in male and female patients with DCM complicated with or without IS**.

Characteristics		Women			Men		
		DCM-IS	non-IS	*p*-value	DCM-IS	non-IS	*p*-value
Numbers of participants		31	130		95	376	
Personal history							
	Age, years	58 (49, 65)	60 (50, 68)	0.604	58 (48, 63)	55 (46, 64)	0.235
	Current smoking (n, %)	3 (9.7)	2 (1.5)	0.077	34 (35.8)	109 (29.0)	0.198
	Current drinking (n, %)	2 (6.5)	3 (2.3)	0.536	28 (29.5)	84 (22.3)	0.145
Past medical history							
	Hyperuricemia (n, %)	7 (22.6)	13 (10.0)	0.108	23 (24.2)	74 (19.7)	0.329
	Hypertension (n, %)	11 (35.5)	38 (29.2)	0.497	46 (48.4)	131 (34.8)	${\mathrm{0.015}}^{*}$
	Systolic blood pressure (mmHg)	119.10 ± 23.14	113.72 ± 17.73	0.095	116 (104, 130)	116.5 (103, 127.8)	0.736
	Hyperlipidaemia (n, %)	7 (22.6)	30 (23.1)	0.953	35 (36.8)	96 (25.5)	${\mathrm{0.028}}^{*}$
	Diabetes (n, %)	6 (19.4)	36 (27.7)	0.342	30 (31.6)	86 (22.9)	0.078
	AF or atrial flutter (n, %)	9 (29.0)	22 (16.9)	0.124	37 (38.9)	103 (27.4)	${\mathrm{0.028}}^{*}$
	NYHA functional class (III, IV) (n, %)	30 (96.8)	87 (66.9)	${\mathrm{0.001}}^{*}$	68 (71.6)	248 (66.0)	0.297
	Left bundle branch block (n, %)	6 (19.4)	44 (33.8)	0.117	16 (16.8)	50 (13.3)	0.374
	Mitral regurgitation (moderate to severe) (n, %)	20 (64.5)	81 (62.3)	0.819	47 (49.5)	215 (57.2)	0.177
Clinical laboratory test							
	Leukocyte (${\mathrm{10}}^{9}$/L)	7.78 ± 2.04	6.63 ± 1.73	0.171	6.55 (5.87, 8.37)	6.85 (5.86, 8.24)	0.412
	Haematocrit (%)	37.5 (34, 42.1)	39.6 (36.2, 41.7)	0.308	42.76 ± 5.06	43.29 ± 4.84	0.835
	Platelets (${\mathrm{10}}^{9}$/L)	218 (172, 261)	214.5 (180, 267)	0.962	196.23 ± 55.04	203.98 ± 57.27	0.692
	Haemoglobin (g/L)	130 (112, 146)	133 (123, 140)	0.279	146.26 ± 18.08	148.95 ± 17.54	0.851
	eGFR (mL/min/1.73 $m^{2}$)	81.1 (59.4, 97.8)	91.2 (72.5, 99.5)	0.146	81.06 (58.18, 95.27)	90.11 (72.46, 102.63)	< ${\mathrm{0.001}}^{*}$
	Serum Na+ (mmol/L)	138.7 (136.6, 140.8)	139.5 (137.5, 140.8)	0.322	138.8 (136, 140.9)	139.8 (137.8, 141.4)	${\mathrm{0.005}}^{*}$
	Hs-CRP (mg/L)	5.8 (1.2, 15.7)	2.06 (0.91, 6.5)	${\mathrm{0.043}}^{*}$	3.04 (0.9, 9.82)	1.77 (0.72, 5.37)	${\mathrm{0.004}}^{*}$
	D-dimer (ng/mL)	227 (100, 643)	165.5 (100, 365.5)	0.505	250 (108, 610)	121 (70.25, 280)	< ${\mathrm{0.001}}^{*}$
Echocardiography							
	LVEDD (mm)	63 (55, 71)	63 (57, 71)	0.635	65 (60, 72)	67.5 (62, 74.8)	0.054
	LVEF (%)	30 (25, 38)	30 (25, 35)	0.818	29 (25, 37)	30 (24, 38)	0.701
	PAP (mmHg)	29 (25, 50)	30.5 (25, 44.3)	0.942	30 (25, 41)	30 (25, 45)	0.300
	LAD (mm)	41 (38, 47)	40 (38, 46)	0.568	46 (41, 51)	47 (41, 51)	0.619
	Intracardiac thrombosis (n, %)	6 (19.4)	5 (3.8)	${\mathrm{0.007}}^{*}$	21 (22.1)	18 (4.8)	< ${\mathrm{0.001}}^{*}$

Data following a normal distribution were presented as the mean ± SD and were analyzed using Student’s *t*-tests. Data following a non-normal distribution were presented as the median (interquartile range) and were analyzed using Mann–Whitney *U* tests. Categorical variables were presented as frequencies (percentages, %) and were analyzed using the chi-squared test. Abbreviations: DCM, dilated cardiomyopathy; IS, ischaemic stroke; AF, atrial fibrillation; NYHA, New York Heart Association; eGFR, estimated glomerular filtration rate; Hs-CRP, hypersensitive C-reactive protein; LVEDD, left ventricular end-diastolic dimension; LVEF, left ventricular ejection fraction; PAP, pulmonary artery pressure; LAD, left atrium diameter; SD, standard deviation; DCM-IS, the DCM with ischaemic stroke group; non-IS, and the DCM without ischaemic stroke group. ^*^ Indicates a significant statistical difference between the two groups.

**Fig. 2.**
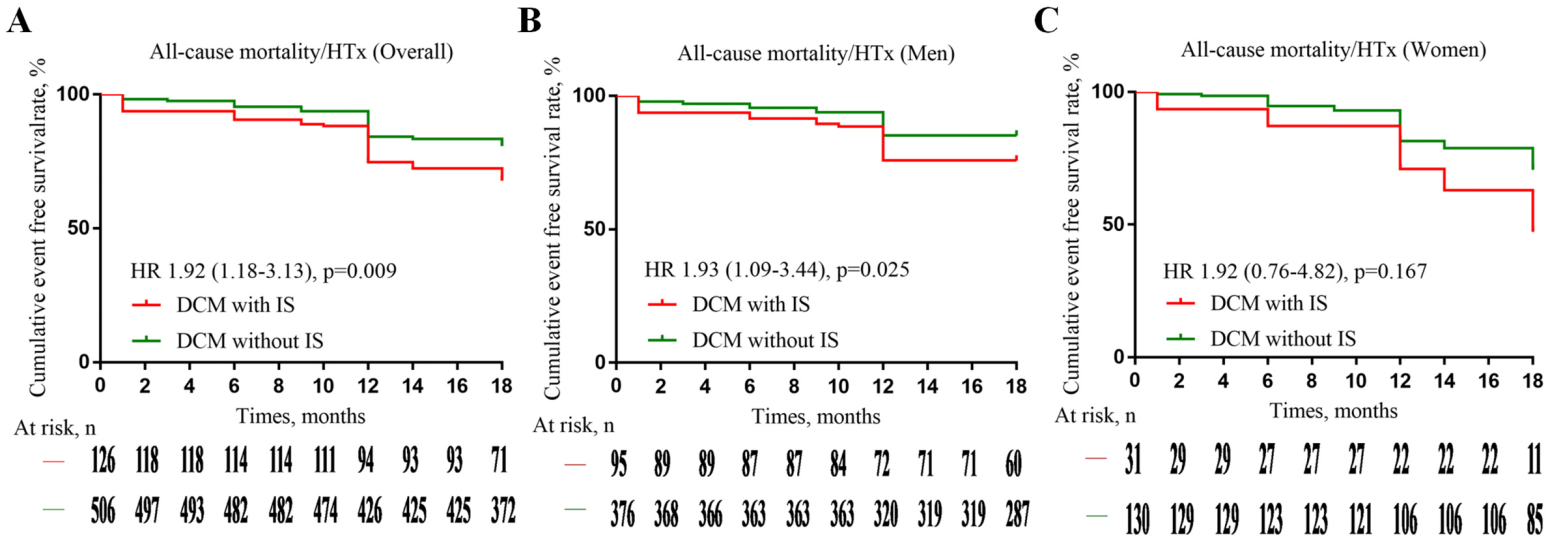
**Kaplan–Meier curves survival of patients with dilated cardiomyopathy complicated with ischaemic stroke (DCM-IS) and without IS (non-IS).** The one-year survival rate was 74.6% in DCM-IS patients and 84.2% in non-IS patients (Figure A). 75.8% in male DCM-IS patients and 85.1% in male non-IS patients (Figure B), as well as 71.0% in female DCM-IS patients and 81.5% in female non-IS patients (Figure C). Abbreviations: HTx, heart transplantation; IS, ischaemic stroke; DCM, dilated cardiomyopathy; HR, hazard ratio.

Overall, patients with 632 DCM underwent at least 1 year (median, 18 (interquartile range: 12–36) months) of follow-up. During the follow-up period, death and HTx were recorded in 74 patients (20 DCM-IS and 54 non-IS cases) and 38 patients (12 DCM-IS and 26 non-IS cases), respectively, with an overall 1-year survival rate of 74.6% in DCM-IS patients and 84.2% in non-IS patients (Fig. 2A). A significant difference in the cumulative survival rate was observed between the two groups (log-rank *p* = 0.009). The causes of death included major ventricular arrhythmias (MVA, including ventricular fibrillation, sustained ventricular tachycardia) (lasting >30 s or with hemodynamic instability), and appropriate ICD interventions (shock or anti-tachycardia pacing on ventricular fibrillation or sustained ventricular tachycardia [18], n = 49), refractory heart failure (HF) (n = 22), and massive cerebral infarction (n = 3). Among the male patients with DCM, death and HTx were recorded in 50 patients (10 cases of DCM-IS, 40 cases of non-IS) and 29 patients (13 cases of DCM-IS, 16 cases of non-IS), respectively, with overall 1-year survival rates of 75.8% in patients with DCM-IS and 85.1% in patients without IS (Fig. 2B). Notably, the cumulative survival rate differed significantly between these two groups (log-rank *p* = 0.025), with the causes of death including MVA (n = 14), refractory HF (n = 34), and massive cerebral infarction (n = 2).

In female patients with DCM, death and HTx were recorded in 24 (5 cases of DCM-IS, 19 cases of non-IS) and 9 patients (4 cases of DCM-IS, 5 cases of non-IS), respectively, with an overall 1-year survival rate of 71.0% in patients with DCM-IS and 81.5% in patients without IS (Fig. 2C); however, no significant difference was observed in the cumulative survival rate when comparing these two groups (log-rank *p* = 0.167). The causes of death included MVA (n = 8), refractory HF (n = 15), and massive cerebral infarction (n = 1).

In the univariate analyses involving all patients with DCM, 18 baseline variables were associated with unfavourable outcomes (all-cause death and HTx); however, lower serum Na+ concentration and LVEF, higher Hs-CRP levels and leukocyte counts, larger LVEDD, worse NYHA functional class, and lack of medication use were retained in the multivariate Cox proportional hazard model (Table 2). Effect modification analyses illustrated significant risk differences in the association between IS status and the poor prognosis in overall patients with DCM by sex (*p* for interaction = 0.073). Subgroup analyses were subsequently conducted. In male patients with DCM, univariate analyses revealed that 13 baseline variables were associated with unfavourable outcomes; however, lower serum Na+ levels, higher leukocyte counts, larger LVEDD, worse NYHA functional class, and lack of medication use were retained in the multivariate Cox proportional hazard model (Table 3). In female patients with DCM, univariate analyses demonstrated that 11 baseline variables were associated with unfavourable outcomes; however, only higher Hs-CRP levels were retained in the multivariate Cox proportional hazards model (Table 4).

**Table 2.** **Factors associated with poor prognosis in patients with DCM within one year (n = 632)**.

Covariates	Univariate analysis		Multivariate analysis	
	HR (95% CI)	*p*-${\mathrm{value}}^{*}$	HR (95% CI)	*p*-value
IS	1.685 (1.118–2.538)	0.013		0.179
Systolic blood pressure at baseline	0.976 (0.965–0.987)	<0.001		0.595
eGFR	0.985 (0.977–0.993)	<0.001		0.194
Serum Na+	0.852 (0.813–0.893)	<0.001	0.925 (0.874–0.978)	0.006^**^
Leukocyte	1.031 (1.007–1.057)	0.012	1.065 (1.021–1.111)	0.003^**^
Hs-CRP	1.016 (1.011–1.020)	<0.001	1.006 (1.000–1.013)	0.048^**^
LVEDD	1.074 (1.056–1.093)	<0.001	1.036 (1.012–1.060)	0.003^**^
LVEF	0.905 (0.881–0.930)	<0.001	0.964 (0.933–0.997)	0.03^**^
PAP	1.028 (1.017–1.039)	<0.001		0.732
LAD	1.049 (1.032–1.067)	<0.001		0.559
Age	0.981 (0.967–0.994)	0.006		0.270
Mitral regurgitation	2.719 (1.743–4.242)	<0.001		0.207
NYHA functional class	6.441 (3.138–13.220)	<0.001	3.257 (1.536–6.909)	0.002^**^
Intracardiac thrombosis	2.109 (1.242–3.580)	0.006		0.989
ACEI/ARB/ARNI	0.514 (0.332–0.796)	0.003	0.525 (0.320–0.863)	0.011^**^
β-Blockers	0.252 (0.131–0.482)	<0.001	0.328 (0.168–0.639)	0.001^**^
ICD/CRT	0.462 (0.241–0.884)	0.020		0.069

Abbreviations: DCM, dilated cardiomyopathy; IS, ischaemic stroke; eGFR, estimated glomerular filtration rate; Hs-CRP, hypersensitive C-reactive protein; LVEDD, left ventricular end-diastolic dimension; LVEF, left ventricular ejection fraction; PAP, pulmonary artery pressure; LAD, left atrium diameter; NYHA, New York Heart Association; ACEI, angiotensin-converting enzyme inhibitor; ARB, angiotensin receptor blocker; ARNI, angiotensin receptor-neprilysin inhibitor; ICD, implantable cardioverter-defibrillators; CRT, cardiac resynchronization therapy; HR, hazard ratios; CI, confidence Interval. ^*^Related factors with statistical differences observed between the group experiencing 1-year outcomes and the controls within the overall DCM group; ^**^Statistical differences were observed after multivariate Cox regression analysis.

**Table 3.** **Factors associated with poor prognosis in male patients with DCM within one year (n = 471)**.

Covariates	Univariate analysis		Multivariate analysis	
	HR (95% CI)	*p*-${\mathrm{value}}^{*}$	HR (95% CI)	*p*-value
IS	1.699 (1.046–2.761)	0.032	1.810 (1.031–3.178)	0.039^**^
eGFR	0.980 (0.970–0.989)	<0.001		0.182
Serum Na+	0.842 (0.793–0.894)	<0.001	0.929 (0.871–0.991)	0.026^**^
Leukocyte	1.029 (1.002–1.057)	0.035	1.069 (1.025–1.115)	0.002
LVEDD	1.088 (1.066–1.110)	<0.001	1.050 (1.022–1.080)	0.001^**^
LVEF	0.906 (0.877–0.935)	<0.001		0.075
PAP	1.034 (1.020–1.048)	<0.001		0.261
LAD	1.054 (1.034–1.073)	<0.001		0.503
Mitral regurgitation	2.833 (1.675–4.793)	<0.001		0.956
NYHA functional class	7.839 (3.169–19.390)	<0.001	3.575 (1.360–9.395)	0.010^**^
ACEI/ARB/ARNI	0.494 (0.296–0.825)	0.007	0.475 (0.272–0.833)	0.009^**^
β-Blockers	0.235 (0.108–0.501)	<0.001	0.324 (0.145–0.724)	0.006^**^
ICD/CRT	0.133 (0.019–0.957)	0.045	0.134 (0.018–0.984)	0.048^**^

Abbreviations: DCM, dilated cardiomyopathy; IS, ischaemic stroke; eGFR, estimated glomerular filtration rate; LVEDD, left ventricular end-diastolic dimension; LVEF, left ventricular ejection fraction; PAP, pulmonary artery pressure; LAD, left atrium diameter; NYHA, New York Heart Association; ACEI, angiotensin-converting enzyme inhibitor; ARB, angiotensin receptor blocker; ARNI, angiotensin receptor-neprilysin inhibitor; ICD, implantable cardioverter-defibrillators; CRT, cardiac resynchronization therapy; HR, hazard ratios; CI, confidence Interval. ^*^Related factors with statistical differences observed between the group experiencing 1-year outcomes and the controls within the overall DCM group; ^**^Statistical differences were observed after multivariate Cox regression analysis.

**Table 4.** **Factors associated with poor prognosis in female patients with DCM within one year (n = 161)**.

Covariates	Univariate analysis		Multivariate analysis	
	HR (95% CI)	*p*-${\mathrm{value}}^{*}$	HR (95% CI)	*p*-value
Systolic blood pressure at baseline	0.977 (0.958–0.997)	0.026		0.894
Serum Na+	0.869 (0.801–0.943)	0.001		0.261
Leukocyte	1.223 (1.033–1.447)	0.019		0.268
Hs-CRP	1.060 (1.031–1.090)	<0.001	1.061 (1.019–1.105)	0.004^**^
LVEDD	1.049 (1.014–1.085)	0.006		0.134
LVEF	0.900 (0.854–0.950)	<0.001		0.152
LAD	1.061 (1.009–1.115)	0.020		0.805
Age	0.974 (0.951–0.998)	0.036		0.359
Mitral regurgitation	2.370 (1.029–5.460)	0.043		0.300
NYHA functional class	4.018 (1.226–13.166)	0.022		0.080
Intracardiac thrombosis	4.964 (2.152–11.452)	<0.001		0.106

Abbreviations: DCM, dilated cardiomyopathy; Hs-CRP, hypersensitive C-reactive protein; LVEDD, left ventricular end-diastolic dimension; LVEF, left ventricular ejection fraction; LAD, left atrium diameter; NYHA, New York Heart Association; HR, hazard ratios; CI, confidence Interval. ^*^Related factors with statistical differences observed between the group experiencing 1-year outcomes and the controls within the overall DCM group; ^**^Statistical differences were observed after multivariate Cox regression analysis.

## 4. Discussion

As a large-scale, multicentre, prospective cohort study, this study demonstrated significantly different baseline characteristics between patients with and without DCM-IS, with a focus on sex disparities. Additionally, it has highlighted the poor short-term prognosis of patients with DCM in the presence of concurrent IS, particularly among men. Furthermore, in male patients with DCM, lower serum Na+ levels, higher leukocyte counts, larger LVEDD, worse NYHA functional class, and lack of medication use were associated with unfavourable outcomes, whereas in female patients with DCM, higher Hs-CRP levels were associated with unfavourable outcomes. Therefore, early identification of IS-related risk factors and timely prevention or intervention could be crucial for improving the prognosis of patients of different sexes.

In recent years, the field of neurocardiology has emerged, focusing on the bidirectional interactions between the heart and brain, encompassing the impact of heart damage on the brain and vice versa [19, 20]. Heart injury often leads to adverse events such as early neurological deterioration and death due to IS [21]. Consequently, heart damage is the second leading cause of acute death after IS and is a crucial determinant of long-term survival [22]. Currently, cardiac disorders such as AF, cardiac ischaemia, heart failure, valvular cardiac disease, patent foramen ovale, and endocarditis are believed to underlie IS pathogenesis [23]. Cardioembolism is considered the primary cause of DCM, involving multiple potential mechanisms, such as haemodynamic disturbance, valvular dysfunction, arrhythmia, or low ejection fraction [12]. In this study, intracardiac thrombosis was notably associated with DCM-IS in both male and female patients, suggesting its role in the pathogenesis of IS in patients with DCM, regardless of sex. Moreover, plasma levels of Hs-CRP have emerged as a valuable biomarker for disease prognosis in patients with intermediate-risk cardiovascular disorders over the past decade. Hence, Hs-CRP could potentially serve for diagnosing and predicting the prognosis of conditions such as acute myocardial infarction, cardioembolic or IS, and hypertrophic or DCM [24, 25]. In alignment with this perspective, our results also revealed higher concentrations of Hs-CRP in both male and female patients with DCM-IS, indicating that routine Hs-CRP measurements might be valuable in identifying high-risk patients with DCM requiring special treatment strategies. Nonetheless, significant differences were also observed in several other variables (hypertension, hyperlipidaemia, AF or atrial flutter, eGFR, serum Na+, and D-dimer) between male and female patients with DCM-IS. This, coupled with prior findings [6, 7, 8, 9], reinforces the notion that sex differences exert a profound influence on IS risk in DCM. However, the exact underlying explanation for this disparity remains enigmatic and warrants future exploration in future research.

Recent evidence has indicated that IS can cause cardiac dysfunction, even in the absence of risk factors and pre-existing heart disease [26]. Although data on the deterioration of cardiac function in DCM following IS remain limited, studies on murine stroke models have reported a progressive 10–25% reduction in LVEF over a period of 3 days to 4 weeks, accompanied by cardiac interstitial fibrosis [27, 28, 29]. A recent meta-analysis of the published studies suggested that compared with female patients, male patients with DCM had an increased risk of all-cause mortality, cardiovascular mortality, and sudden cardiac death [25]. Another study by Cocker *et al*. [30] reported that among patients with acute myocarditis, male patients were twice as likely as female patients to present with myocardial fibrosis. In this study, patients with DCM with concurrent IS experienced poor short-term outcomes, especially in men. This supports the idea that IS can, at least partially, exacerbate cardiac dysfunction and even result in death, particularly in male patients.

Currently, male sex is considered a significant risk factor for developing heart failure in several cardiovascular conditions, including DCM. Sex hormones may play a role in the progression of DCM, as evidence suggests their influence on sex-specific molecular mechanisms, glucose and lipid metabolism, cardiac energy metabolism, and function [31]. Notably, multiple animal studies have revealed that testosterone can lead to increased cardiac inflammation, remodelling, and DCM by interacting with androgen receptors on cardiac vascular endothelial cells, smooth muscle cells, fibroblasts, and myocytes [6, 32, 33]. Furthermore, inflammatory cell and cytokine activation occurs more often in men, leading to complement and platelet activation, thereby predisposing patients with DCM to thromboembolic events, such as stroke [34, 35]. Consistent with these findings, our results showed significant associations between lower serum Na+ levels, higher leukocyte counts, larger LVEDD, worse NYHA functional class, and unfavourable outcomes in male patients with DCM-IS. Conversely, only higher Hs-CRP levels were linked to poor prognosis in female patients with DCM-IS, although numerous studies suggested that elevated baseline Hs-CRP levels serve as clinically significant biological markers for poor prognosis in IS patients [36]. This odd phenomenon was deemed to result from a low positive sample size (n = 31/161) and higher covariates (n = 11) in the multivariate Cox proportional hazard mode, which made the results relatively less reliable. However, all independent variables were rigorously screened before inclusion in the multifactorial Cox regression analysis. Besides, the hazard ratios (HR) and confidence intervals derived from the Cox regression analysis are within the expected normal range. While acknowledging a degree of fragility in the results, the observed characteristics align reasonably well with similar metrics from prior studies, thereby lending a degree of reliability to our findings. To sum up, these findings underscore the role of sex-specific cardiac physiology or cardiometabolic conditions in the clinical outcomes of patients with DCM. However, our findings should be interpreted with caution, especially owing to the smaller sample size of female patients with DCM, which may have introduced bias to the results. Therefore, larger cohort studies with a more balanced distribution of sexes are necessary for further validation.

This study has some limitations. Firstly, the primary limitation is the lack of statistical power owing to the relatively small sample size, particularly among female patients. Secondly, a selection bias may have occurred due to disparities in patient enrolment between the three medical centres. Finally, the follow-up period was relatively short for most patients. Furthermore, the high loss-to-follow-up rate was one of the shortcomings of this study. Future improvements could involve optimizing follow-up methods and enhancing patient services. Nevertheless, the response rate in this study still reached 76.0% (632/830), indicating a certain level of reliability in the conclusions.

## 5. Conclusions

To the best of our knowledge, this is the first study to investigate the impact of concurrent IS on the prognosis of male and female patients with DCM. Patients with DCM have a poor prognosis when IS is present concurrently, particularly among male patients. Multiple variables, including serum Na+ levels, leukocyte count, LVEDD, and NYHA functional class were identified as risk factors for unfavourable outcomes among male patients with DCM-IS within 1 year, in comparison with their female counterparts. Therefore, emphasis should be placed on the occurrence of IS in patients with DCM. Early and proactive secondary prevention of cerebrovascular diseases may improve the prognosis of DCM patients. Intervention studies focusing on male patients with DCM complicated with IS should be prioritised. Nonetheless, prospective randomised controlled studies with longer follow-up periods in carefully selected cases are required to confirm our findings.

## Data Availability

The datasets used and/or analyzed during the current study are available from the corresponding author on reasonable request.

## Abbreviations

DCM, dilated cardiomyopathy; IS, ischaemic stroke; AF, atrial fibrillation; LVEF, left ventricular ejection fraction; LVFS, left ventricular fractional shortening; LVEDD, left ventricular end-diastolic dimension; eGFR, estimated glomerular filtration rate; Hs-CRP, hypersensitive C-reactive protein; NYHA, New York Heart Association; HF, heart failure; PAP, pulmonary artery pressure; LAD, left atrium diameter; ACEI, angiotensin-converting enzyme inhibitor; ARB, angiotensin receptor blocker; ARNI, angiotensin receptor-neprilysin inhibitor; ICD, implantable cardioverter-defibrillators; CRT, cardiac resynchronization therapy.
